# Adverse neonatal outcomes and associated factors among births through operative vaginal delivery at the University of Gondar Comprehensive Specialized Hospital, Northwest Ethiopia: A facility-based retrospective cross-sectional study

**DOI:** 10.1371/journal.pone.0357043

**Published:** 2026-08-28

**Authors:** Shambal Kassahun Ferrede, Mulatu Werkina Eticha, Habtamu Molla Ayele

**Affiliations:** 1 Department of Obstetrics and Gynecology, Bedele General Hospital, Buno Bedele Zone, Ethiopia; 2 Department of Midwifery, Arjo Primary Hospital, East Wollega Zone, Ethiopia; 3 Maternal and Child Health Directorate, Federal Ministry of Health, Addis Ababa, Ethiopia; University of Oulu: Oulun Yliopisto, FINLAND

## Abstract

**Background:**

Obstetric interventions can improve maternal and neonatal outcomes when performed appropriately by skilled healthcare professionals. Appropriate use of forceps and vacuum extraction can reduce neonatal complications associated with operative vaginal deliveries. However, there is limited evidence on the prevalence of adverse neonatal outcomes and associated factors among operative vaginal deliveries in Northwest Ethiopia.

**Objective:**

This study aimed to assess the prevalence of adverse neonatal outcomes and associated factors among births through operative vaginal delivery at the University of Gondar Comprehensive Specialized Hospital in Northwest Ethiopia.

**Methods:**

A facility-based retrospective cross-sectional study was conducted among 303 mothers who underwent operative vaginal delivery at the University of Gondar Comprehensive Specialized Hospital from September 1, 2019, to August 30, 2021. Data was extracted using a structured questionnaire. The collected data was entered into Epi-Data version 3.1 statistical software and exported to IBM SPSS statistics version 24 for data analysis. Descriptive statistics were used to summarize the participants’ characteristics and neonatal outcomes. Bivariable logistic regression analysis was performed to identify candidate variables. Variables with a p-value < 0.25 were entered into the multivariable logistic regression model. Adjusted odds ratio (AOR) with 95% confidence intervals (CIs) were calculated to assess the strength of associations. Statistical significance was declared at a p-value of < 0.05 in the multivariable logistic regression analysis.

**Results:**

The prevalence of adverse neonatal outcomes among births delivered through operative vaginal delivery was 23.1% (95% CI: 18.68–28.21). The mean age and standard deviation of the study participants were 25.19 ± 4.7 years. All participants were married and 154 (50.8%) had obtained a college education or higher. The presence of grade ½ (AOR = 4.13, 95% CI: 1.96–8.69) and grade three meconium-stained amniotic fluid (AOR = 24.9, 95% CI: 6.14–46.06) and primigravidity (AOR = 2.49, 95% CI: (1.13–5.16) were significantly associated with adverse neonatal outcomes.

**Conclusions:**

Adverse neonatal outcomes among births through operative vaginal delivery at the University of Gondar Comprehensive Specialized Hospital were high. Close intrapartum monitoring and early obstetric intervention among women with meconium-stained amniotic fluid and primigravid mothers are essential to reduce adverse neonatal outcomes associated with operative vaginal delivery.

## Introduction

Operative vaginal delivery (OVD) refers to a vaginal birth assisted by forceps or vacuum devices during the second stage of labor to expedite delivery when maternal or fetal indications are present [1–4]. It is an important obstetric intervention that can improve maternal and neonatal outcomes when performed appropriately by skilled healthcare professionals. Globally, 10–20% of all deliveries may require some form of intervention and operative vaginal delivery accounts for 2–23% of all deliveries. In the United Kingdom, the OVD ranges from 10% to 15%. However, studies conducted in Ethiopia have reported rates ranging from 7% to 16.9% [5–8].

Operative vaginal deliveries are performed for the indication of maternal or fetal-related conditions and any occasion that threatens the mother or fetal life [9]. Commonly reported indications for OVD are prolonged second stage, non-reassuring fetal heart rate patterns maternal exhaustion and medical conditions requiring shortening of the second stage of labor [10,11]. Despite its clinical benefits, operative vaginal delivery remains associated with several neonatal complications. Minor neonatal complications following OVD include soft tissue trauma, cephalohematoma, jaundice, and transient brachial plexus injury while major complications include hypoxic ischemic encephalopathy, intracranial and sub-galeal hemorrhage, seizures, cranial fracture, permanent brachial plexus injury, admission to the neonatal intensive care unit (NICU) and death [12–14]. Furthermore, failed operative vaginal delivery and repeated instrument application may increase the risk of neonatal morbidity, including low Apgar scores, need for intubation, prolonged stay in the neonatal unit, hospitalization, and neurologic complications(seizures) [15,16]. Neonates delivered by operative vaginal delivery with an indication of Non-Reassuring Fetal Heart Rate (NRFHRP) had more likely to have poor Apgar scores than those with indications of poor maternal effort [7,8].

Ethiopia has introduced different strategies to reduce neonatal morbidity and mortality and achieve the sustainable development goal’s target by 2030; however, the rates remain high. Existing studies have largely focused on adverse birth outcomes following spontaneous vaginal deliveries with limited attention given to operative vaginal delivery despite its potential for both neonatal benefits and harm. Knowing the prevalence of adverse neonatal outcomes and identifying possible risk factors for complications related to operative vaginal delivery may impact the use of the procedure by health professionals. Therefore, this study aimed to assess the prevalence of adverse neonatal outcomes and associated factors among births through operative vaginal delivery at the University of Gondar Comprehensive Specialized Hospital in Northwest Ethiopia.

## Materials and methods

### Study area and period

The study was conducted at the University of Gondar Comprehensive Specialized Hospital (UoGCSH) in Gondar, the capital of the North Gondar administrative zone, approximately 741 km northwest of Addis Ababa and 172 km northeast of Bahir Dar. The University of Gondar Comprehensive Specialized Hospital is one of the oldest hospitals in Ethiopia. It provides a range of specialties, such as gynecology and obstetrics, pediatrics, surgery, internal medicine, psychiatry, and HIV care. The hospital has more than 1,040 healthcare professionals, 580 beds in five inpatient departments and 14 wards, and outpatient services provided in 14 different units. The gynecology and obstetrics department unit has gynecologists, obstetricians, nurses, midwives and resident physicians. The Department of Gynecology and Obstetrics runs one labor and delivery ward with nine beds in the first-stage room, six delivery couches in the second-stage room along with two emergency operating rooms, three postpartum maternity wards, one high-risk ward, one gynecology ward, one urogynecology ward, four gynecologic outpatient departments (OPD), four antenatal clinics, and Michu clinic. A total of 19,634 deliveries were recorded over the two years from September 1, 2019 to August 30, 2021. Of which, 719 (3.66%) were by operative vaginal deliveries, 5594 (28.49%) were by cesarean section and 13321 (67.85%) were vaginal deliveries.

### Study design

A facility-based retrospective cross-sectional study design was conducted.

### Source population

All mothers who underwent operative vaginal delivery at the University of Gondar Comprehensive Specialized Hospital in Northwest Ethiopia.

### Study population

All mothers who underwent operative vaginal delivery at the University of Gondar Comprehensive Specialized Hospital and fulfilled the inclusion criteria during the study period were included.

### Inclusion criteria

All mothers who underwent operative vaginal delivery (forceps and/or vacuum devices) at the University of Gondar Comprehensive Specialized Hospital from September 2019 to August 2021 were included in the study.

### Exclusion criteria

Mothers whose deliveries resulted in stillbirths, neonates with lethal congenital anomalies, and multiple births (twins or more) were excluded. Medical records with incomplete documentation of key study variables were also excluded.

### Sample size determination

The sample size was estimated using a single population proportion formula. By considering a 95% confidence level, 4% margin of error and prevalence of adverse neonatal outcomes of operative vaginal delivery was taken as 13.2% as obtained from a study conducted at Jimma University Medical Centers, Southwest Ethiopia [7].

The required sample size was calculated using the following single-population proportion formula:


$$\begin{array}{c} n=\frac{{\left(\frac{Za}{2}\right)}^{2}p\left(1-p\right)}{d^{2}} \end{array}$$

Were,

P = Adverse neonatal outcome of OVD (13.2%),

d = Margin of tolerated sample error (4%) and

Zα/_2_= The standard normal distribution at 1- α/_2_% confidence level (95%=1.96)

n: Sample size,


$$\begin{array}{c} \text{n }=\;\frac{(\text{Za}{/}_{\text{2}}{)}^{\text{2}}\text{ p}(\text{1}-\text{p})\;}{d^{\text{2}}}=\;\frac{(\text{1}.\text{96}{)}^{\text{2}}*\text{0}.\text{132}*\text{0}.\text{868}}{{\left(\text{0}.\text{0}\text{4}\right)}^{\text{2}}}\;=\;\frac{\text{0}.\text{4401551616}}{\text{0}.\text{0}\text{0}\text{1}\text{6}}\;=\text{ 275} \end{array}$$

After adding a 10% non-response rate, the final sample size was 303 participants.

### Sampling Technique

At the University of Gondar Comprehensive Specialized Hospital, 19634 deliveries were recorded over two years from September 1, 2019, to August 30, 2021, of which 719 neonates were delivered by operative vaginal delivery, 5594 were delivered by cesarean section, 13321 were vaginal deliveries. A total of 719 operative vaginal deliveries were identified during the study period, and 303 operative vaginal deliveries were selected using computer-generated simple random sampling technique (Fig 1).

### Data collection procedures

The data collection instrument was a semi-structured questionnaire adapted from previous studies [5,6,9] (S1 File). The questionnaire was validated through a pilot study conducted at a nearby health center to check for the validity of questionnaire. A structured tool was prepared in English, translated into the local language of Amharic, and then translated back into English to check for consistency. The data collectors were professional midwives and medical interns who worked in the labor and delivery wards. Onsite training was provided to the data collectors on the methods of collecting data by reviewing the patient medical records (charts). The training also focused on how to fill out the questionnaire and the ethical aspects of maintaining the confidentiality of the information.

### Variables

**Fig 1:**
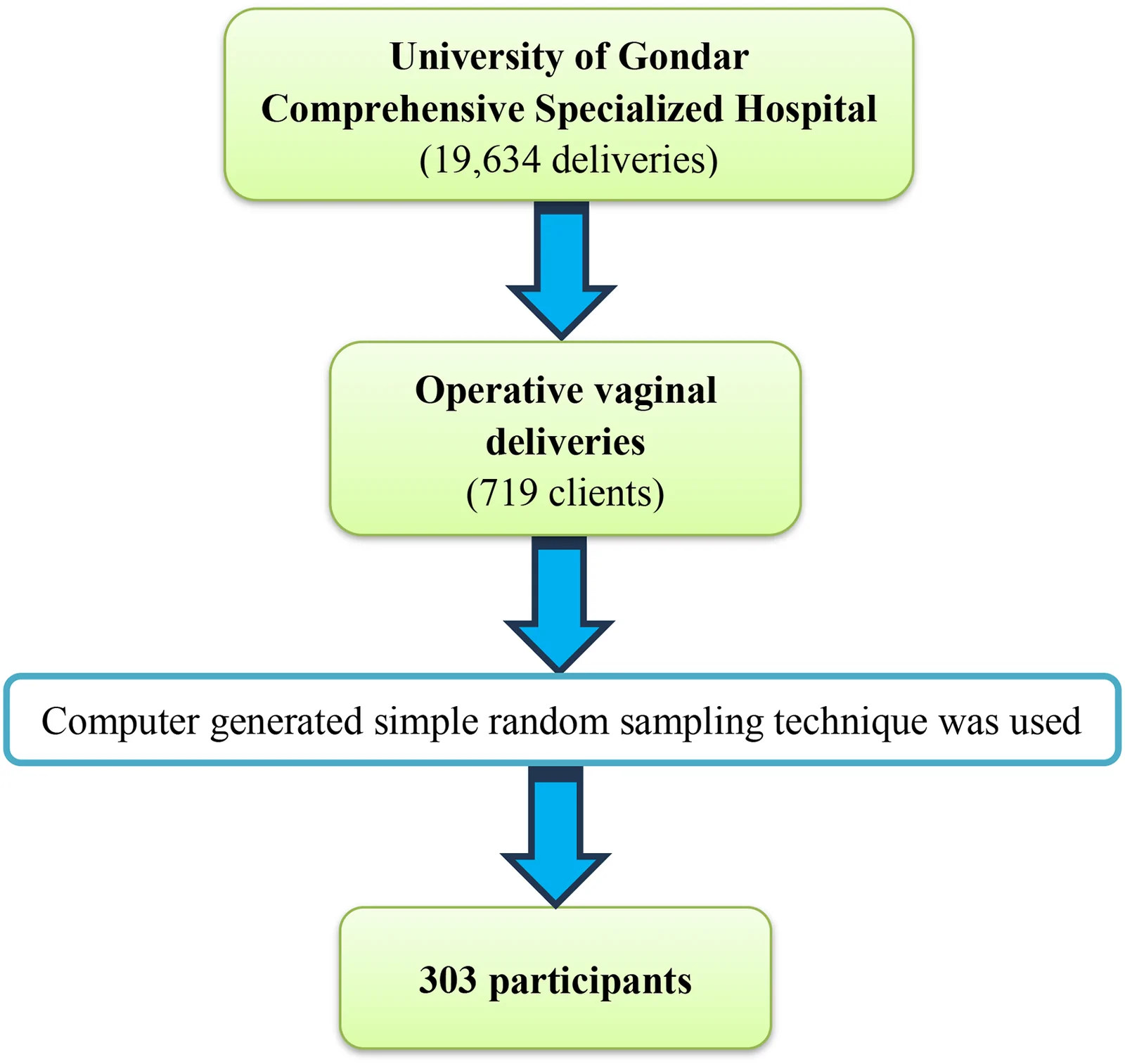
Sampling procedure of prevalence of adverse neonatal outcomes and associated factors among births through operative vaginal delivery at the University of Gondar Comprehensive Specialized Hospital, Northwest Ethiopia.

### Dependent variable

- Adverse neonatal outcomes of operative vaginal delivery

### Independent variables

- **Socio-demographic factors**: Age, sex, residency, educational status, occupation, ANC follow-up and marital status.
- **Obstetric and medical-related factors**: Gravidity (number of pregnancies), history of previous abortion, gestational age at the time of delivery, bad obstetric history (history of stillbirth or early neonatal death), parity (number of previous viable deliveries), neonatal weight (weight of the neonate after delivery), fetal heart rate pattern (the fetal heart rate condition during the intrapartum course), underlying medical condition (diabetes mellitus, hypertension, cardiac illness), liquor status (presence of meconium-stained amniotic fluid), duration of second stage, experience of the operator, presence of other obstetric complications (antepartum hemorrhage, preeclampsia, (premature rupture of membrane, chorioamnionitis), type of instrument used (vacuum or forceps), type of operative vaginal delivery applied (outlet or low), time of operative vaginal delivery applied (followed in the ward or on arrival), station of the presenting part at the time of the procedure, episiotomy and stage of labor at the time of admission (first stage or second stage).
- **Health facility and healthcare provider-related factors:** Health worker profession, distance from the hospital, time to reach the hospital and waiting time for gate service.

### Operational definitions

**Operative vaginal delivery:** The operator uses forceps or a vacuum device to assist the mother during the second stage of labour and reduce unnecessary cesarean section deliveries [6].

**APGAR score**: Acronym for appearance, pulse rate, grimace reflex, activity and respiratory rate which is used to assess the health status of newborns within the first and fifth minutes after birth [17].

**Birth Trauma**: Birth trauma is any trauma to the newborn as a result of labor and delivery like cephalhematoma, sub-galeal hemorrhage, retinal hemorrhage, shoulder dystocia, clavicle fracture, and scalp lacerations [7,8,18].

**Adverse neonatal outcomes of operative vaginal delivery**: This is considered when the neonates experience one or more of the following complications: low APGAR score, retinal hemorrhage, anemia, need for resuscitation, admission to NICU, neonatal birth trauma and neonatal death [17,19].

**Immediate birth outcomes** are the immediate maternal or neonatal conditions that could be complicated or non-complicated occurred within the first six hours of delivery [6].

**Non Reassuring Fetal Heart Rate:** This is defined as when the fetal hear rate is less than 100 or higher than 180 beats per minute [20].

### Data quality control

To maintain data quality, the questionnaires prepared using the English version were translated into Amharic and back into English to check for consistency. A pre-test was conducted to check the precision and clarity of the questions on 5% of the total sample size. Based on the pre-test, necessary corrections and modifications were made before starting the actual study. The data collectors and supervisors were trained prior to data collection. The completeness of the questionnaires and the overall quality of data collection were monitored daily by the supervisor and principal investigator. The data collected was rechecked before starting the data analysis.

### Data processing and analysis

The data were checked for quality and consistency. The data were then entered into Epi data version 3.1 and exported to SPSS 24 for statistical analysis. Furthermore, data cleaning (editing, recording and checking for missing values) was performed after exporting to SPSS. Descriptive statistics were explained using frequencies and percentages. Means and standard deviations were used to describe continuous variables. Multicollinearity was assessed using variance inflation factor and tolerance values. The results showed no evidence of multicollinearity. The association between adverse neonatal outcomes and associated factors was assessed using bivariable logistic regression analysis. Variables with a p-value < 0.25 in the bivariable logistic regression analysis were entered into the multivariable analysis regression model. Multivariable analysis was performed to determine the presence of statistically significant associations between independent and dependent variables at p-value < 0.05. The Hosmer-Lemeshow goodness of fit test was used to assess how the data fit the model. Adjusted Odds Ratio and 95% Confidence Intervals were calculated and reported to determine statistically significant associations between the dependent and independent variables.

### Declarations

#### Ethical Considerations.

Ethical approval was obtained from the Ethical Review Committee of the Institutional Review Board of the University of Gondar (Ref. No: 642/2021). With this clearance, formal approval was sent and permission to conduct the study was secured from the administration of Gondar University Comprehensive Specialized Hospital and the head of the Department of Gynaecology and Obstetrics before commencing the study. After the study was confirmed, data related to the study participants were obtained from Gondar University Compressive Specialized Hospital in an anonymized format and the Ethical Review Committee of the Institutional Review Board of the University of Gondar waived the requirement for informed consent. The datasets were de-identified at the source by the trained personnel. The study team was committed to using the data only for this study, and no one outside the study team had access to the data.

## Results

### Socio-demographic characteristics of the study participants

A total of 303 mothers who underwent operative vaginal delivery were included in this study. The mean age and standard deviation (±SD) of the study participants were 25.19 ± 4.7 years. Nearly two-thirds of the participants, 191(63%) were from Gondar Town. Of all participants, 154 (50.8%) had obtained a college or higher educational levels (Table 1).

**Table 1:** Socio-demographic characteristics of mothers who gave birth by operative vaginal delivery in the University of Gondar Comprehensive Specialized Hospital, September 2021 (N = 303).

Variables	Frequency (#)	Percent (%)
**Age (years)**		
15-19	20	6.6
20-24	107	35.3
25-29	130	42.9
≥30	46	15.2
**Residence**		
Gondar Town	191	63
Outside Gondar Town	112	37
**Marital status**		
Single	0	0
Married	303	100
**Educational status**		
No formal education	11	3.6
Primary level (1–8 grade)	51	16.9
Secondary level (9–12 grade)	87	28.7
College and above	154	50.8
**Occupation**		
Farmer	71	23.4
Merchant	117	38.7
Private employee	14	4.6
Governmental employee	101	33.3

### Obstetric history of the study participants

According to this study, 234 (77.2%) were primiparous and 69 (22.8%) of them were multiparas. All mothers had attended at least one Antenatal Care (ANC) visit. The most common indication for operative vaginal delivery was a prolonged second stage of labor accounting for 215 (71%) cases, followed by non-reassurance fetal heart pattern (NRFHRP), 81 (26.7%). Among the types of operative vaginal deliveries, forceps were more commonly used in 175 (57.8%) participants, whereas vacuum deliveries were performed in 128 (42.2%) cases. Approximately 72 (23.8%) of the women had different grades of MSAF (Table 2).

**Table 2:** Obstetric history of the mothers who gave birth by operative vaginal delivery in the University of Gondar Comprehensive Specialized Hospital, September 2021 (N = 303).

Characteristics	Frequency (#)	Percent (%)
**Parity**		
1	234	77.2
2-4	62	20.5
≥5	7	2.3
**Gestational age**		
Preterm	21	6.9
Term	259	85.5
Post term	23	7.6
**Type of OVD used**		
Vacuum	128	42.2
Forceps	175	57.8
**Type of OVD applied**		
Low	63	20.8
Outlet	240	79.2
**Time of application**		
On arrival	44	14.5
Followed	259	85.5
**Status of liquor**		
Clear	231	76.2
Grade 1 MSAF	25	8.3
Grade 2 MSAF	26	8.6
Grade 3 MSAF	21	6.9
**Weight of the newborn in grams**		
1500-2499	16	5.3
2500-3999	283	93.4
≥ 4000	4	1.3
**Delivery conducted by**		
Midwives	110	36.3
Gynecologists	115	38
Emergency surgeons	78	25.7

### Adverse neonatal outcomes among births through operative vaginal delivery

Of all births through operative vaginal delivery, the prevalence of adverse neonatal outcomes was 70 (23.1%; 95% CI: 18.68–28.21). The prevalence of adverse neonatal outcomes was higher among primigravida mothers (24.4%) and in post-term births (26.1%). Based on indications, NRFHRP had a higher proportion of adverse neonatal outcomes (39.9%) than the prolonged second stage of labor (16.27%). The most common adverse outcome was neonatal resuscitation (19.5%) followed by NICU admission (18.5%) (Table 3).

**Table 3:** Adverse neonatal outcomes among births through operative vaginal delivery in the University of Gondar Comprehensive specialized hospital, September 2021 (N = 303).

Variable	Adverse neonatal outcomes		Total
	Yes (# (%))	No (# (%))	
**Age**			
15-19	5(25)	15(75)	20(100)
20-24	27(25.2)	80(74.8)	107(100)
25-29	29(22.3)	101(77.7)	130(100)
≥30	9(25)	27(75)	36(100)
**Place of the residency**			
Gondar Town	37(19.4)	154(80.6)	191(100)
Outside Gondar Town	33(29.5)	79(70.5)	112(100)
**Parity**			
1	57(24.4)	177(75.6)	234(100)
2-4	12(19.4)	50(80.6)	62(100)
≥ 5	1(14.3)	6(85.7)	7(100)
**Gestational age**			
Preterm	3(14.3)	18(85.7)	21(100)
Term	61(23.6)	198(76.4)	259(100)
Post term	6(26.1)	17(73.9)	23(100)
**Type of OVD used**			
Vacuum	22(17.2)	106(82.8)	128(100)
Forceps	48(27.4)	127(72.6)	175(100)
**Type of OVD applied**			
Low	20(31.7)	43(68.3)	63(100)
Outlet	50(20.8)	190(79.2)	240(100)
**Time of application**			
On arrival	20(45.5)	24(54.5)	44(100)
Followed	50(19.3)	209(80.7)	259(100)
**Status of liquor**			
Clear	30(13)	201(87)	231(100)
Grade 1 MSAF	7(18.2)	18(81.8)	25(100)
Grade 2 MSAF	15(56.7)	11(43.3)	26(100)
Grade 3 MSAF	18(85.7)	3(14.3)	21(100)
**Weight of the newborn in grams**			
1500-2499	12(75)	4(25)	16(100)
2500-3999	219(86.6)	64(13.4)	283(100)
≥4000	2(50)	2(50)	4(100)

*Preterm= GA< 37 weeks, Term= GA= 37- 41 weeks, Post term= GA ≥ 42 weeks

### Factors associated with adverse neonatal outcomes among operative vaginal deliveries

In the bivariable logistic regression analysis, variables such as gravidity, stage of labor at the time of admission, status of the membrane at the time of admission, status of liquor, place of residence, indication for operative vaginal delivery, experience of the operator, type of instrument used for operative vaginal delivery, type of operative vaginal delivery applied, episiotomy, duration of the second stage and time of application were identified as candidate variables at a p-value of < 0.25. A multivariable logistic regression model was used to identify independent factors affecting neonatal outcomes. After controlling for the possible effects of confounding variables in the final multivariable analysis model, grade ^1^/_2_ MSAF, grade 3 MSAF and neonates born to primigravida mothers were significantly associated with adverse neonatal outcomes.

The odds of adverse neonatal outcomes among births with grade ^1^/_2_ MSAF and grade 3 MSAF were 4.13 times (AOR = 4.13, 95% CI: 1.96–8.69) and 24.9 times (AOR = 24.90, 95% CI: 6.14–46.06) higher than those with clear liquor status. Neonates born to primigravida mothers had 2.49 times (AOR = 2.49, 95% CI: 1.13–5.16) higher odds of adverse neonatal outcomes than neonates born to multigravida mothers (Table 4).

**Table 4:** Bivariable and multivariable logistic regression analysis of factors associated with adverse neonatal outcomes in the University of Gondar Comprehensive Specialized Hospital, September 2021 (N = 303).

Variables	COR (95% CI)	AOR (95% CI)	P-value
**Residency**			
Urban	Ref	Ref	
Rural	1.74(1.01 - 2.99)	0.70 (0.36 - 1.35)	0.29
**Gravidity**			
Multigravida	Ref	Ref	
Primigravida	2.06(1.08 - 3.91)	2.49(1.13 - 5.16)	**0.03** ^*^
**Indication for OVD**			
Prolonged 2^nd^ stage of labour	Ref	Ref	0.26
NRFHRP	3.39(1.94 - 5.94)	0.59(0.25 - 1.45)	
**Type of OVD used**			
Outlet	Ref	Ref	
Low	1.77(0.96 - 3.27)	0.79(0.35 - 1.84)	0.67
**Time of application**			
Followed	Ref	Ref	
On arrival	3.48(1.76 - 6.79)	0.56(0.12 - 2.63)	0.67
**Type of instrument**			
Vacuum	Ref	Ref	
Forceps	1.82(1.03 - 3.21)	1.29(0.64 - 2.63)	0.47
**Status of liquor**			
Clear	Ref	Ref	
Grade ½	5.08(2.59 - 9.97)	4.13(1.96 - 8.69)	**0.00** ^*^
Grade 3	40.2(11.17 - 114.74)	24.90(6.14 - 46.06)	**0.02** ^*^
**Stage of labor at admission**			
First stage	Ref	Ref	
Second stage	3.16(1.71 - 5.85)	1.25(0.30 - 5.16)	0.83
**Membrane status at admission**			
Intact	Ref	Ref	
Ruptured	2.42(1.33 - 4.44)	1.67(0.81 - 3.46)	0.16
**Duration of second stage**			
< 2 hours	Ref	Ref	
≥ 2 hours	0.31(0.17 - 0.57)	0.66(0.24 - 1.81)	0.63
**Episiotomy**			
Yes	Ref	Ref	
No	0.15(0.02 - 1.11)	0.20(.002 - 1.86)	0.23
**Delivery conducted by**			
Midwives	0.29(0.67 - 1.320)	0.49(0.07 - 3.41)	0.47
Gynecologists	0.33(0.13 - 0.80)	0.39(0.14–1.140)	0.08
Emergency surgeons	Ref	Ref	

1= reference point. *= statistically significant, p-value < 0.05; CI = Confidence Interval; COR = Crude Odds Ratio; AOR = Adjusted Odds Ratio

## Discussion

This study aimed to determine the prevalence of adverse neonatal outcomes and associated factors among births through operative vaginal delivery at the University of Gondar Comprehensive Specialized Hospital, Northwest Ethiopia. The prevalence of adverse neonatal outcomes was 23.1% (95% CI: (18.68% − 28.21%). The prevalence of adverse neonatal outcomes was higher among primigravida mothers (24.4%) and in post-term births (26.1%). The prevalence in this study is consistent with a study conducted at Aksum Saint Marry Hospital (20%) and a study conducted at Arba-minch General Hospital (24%) and a study conducted at a tertiary hospital in Nepal (25%) [17,21,22].

The result of this study is higher than that reported in a study conducted at Felegehiwot Comprehensive Specialized Hospital (12.1%) [23], Jimma University Medical Center (13.2%) [7], Nigist Eleni Mohammed Memorial Comprehensive Specialized Hospital (19%) [24]. This discrepancy might be due to the comprehensive specialized hospital where a high number of complicated neonatal and maternal cases were referred from other hospitals and health centers to this hospital. However, this result is lower than a study conducted at Dilla University Referral Hospital (42.1%) and Nigeria (31%) [8,25]. The difference could be due to the availability of trained staff, operative equipment and access to cesarean section delivery at the hospital.

In this study, MSAF was significantly associated with adverse neonatal outcomes and the odds of adverse neonatal outcomes among births with Grade 1/2 MSAF and Grade 3 MSAF were 4.13 and 24.9 times respectively, compared to births with clear liquor status, which is consistent with the findings of other studies. A study conducted at Addis Ababa, Jimma University Medical Center and Dilla University referral hospital showed that, the most common indication was fetal distress (NRFHRP) due to the presence of meconium [7,17,26]. This can be explained by the fact that the passage of meconium is often a response to fetal compromise, increasing the risk of meconium aspiration syndrome, respiratory distress, neonatal resuscitation and NICU admissions.

This study also showed that primigravidity was 2.49 times more likely to be associated with adverse neonatal outcomes as compared to multigravida. This finding is consistent with those studies conducted in Canada and Nepal [9,27]. A possible reason for this is that primigravida women may experience a longer second stage of labor, which can lead to complications such as fetal distress or the need for assistance. Notably, according to our study results, a prolonged second stage of labor was the most common indication for operative vaginal delivery (71%). This study is consistent with studies conducted in India and Nepal [9,28]. This may not be truly attributable to the procedure, as adverse neonatal outcomes may be the outcome of the events of labor that indicated the intervention rather than operative vaginal delivery itself [28].

### Limitation of the study

This study had several limitations. First, the retrospective design of this study missed some important variables limiting the establishment of a causal relationship. Second, the cross-sectional nature of the study precluded the establishment of temporal relationships between exposure and outcome variables. Third, the study was conducted at a single comprehensive specialized hospital, which limits the generalizability of the study.

## Conclusions

The prevalence of adverse neonatal outcomes among births delivered through operative vaginal delivery was high. The presence of grade ½, and grade three meconium-stained amniotic fluid and primigravidity were the determinant factors for adverse neonatal outcomes among operative vaginal deliveries.

### Recommendations

#### To the hospital and health workers.

Most mothers for whom operative vaginal delivery was performed on arrival and who had adverse neonatal outcomes were referred from other hospitals and health centers. Operative vaginal delivery did not require referral to a tertiary care hospital. Provision of high-quality antenatal care, close monitoring of primigravida and labouring women and timely interventions should be a priority to decrease adverse neonatal outcomes.

#### To Ministry of Health and policy makers.

Strengthening healthcare workers skill in operative vaginal delivery, establishing a well-structured health facility referral system and increasing health facility access significantly improve neonatal outcomes. In addition, health facilities should be equipped with the instruments needed for operative vaginal deliveries.

#### To researchers.

Further studies using primary data should be conducted to investigate the risks and benefits of operative vaginal delivery.

## Supporting information

S1 File Questionnaires. (DOCX)

S2 File Summary of datasets. (XLSX)

## Abbreviations

ANC: Antenatal Care
APGAR: Appearance, Pulse, Grimace, Activity and Respiration
AOR: Adjusted Odds Ratio
CI: Confidence Interval
MSAF: Meconium-Stained Amniotic Fluid
NRFHR: Non-Reassuring Fetal Heart Rate
NICU: Neonatal Intensive Care Unit
OVD: Operative Vaginal Delivery
SPSS: Statistical Package for Social Science
UoGCSH: University of Gondar Comprehensive Specialized Hospital
