# Supplementary material for: Adverse neonatal outcomes and associated factors among births through operative vaginal delivery at the University of Gondar Comprehensive Specialized Hospital, Northwest Ethiopia: A facility-based retrospective cross-sectional study
Source: PLoS One. 2026 Aug 28;21(8):e0357043. doi: 10.1371/journal.pone.0357043 (PMC13524286; doi:10.1371/journal.pone.0357043)
Supplement: Supplementary file 1 [file pone.0357043.s005.pdf]

PONE-D-25-28741R1

Adverse neonatal outcomes and associated factors among births through operative vaginal delivery at the University of Gondar Comprehensive Specialized Hospital, Northwest Ethiopia: A facility based retrospective cross-sectional study

Thank you so much the editor and reviewers of your valuable comments. We saw these comments as an input for having a better manuscript.

Reviewers' comments and our responses to questions

Reviewer #3:

Abstract

Background:

Too general or lacks quantitative context

Add specific statistics and not write its burden in % in abstract part

Response:

Thank you very much the reviewer for your valuable comment. The comment is accepted and updated within the manuscript from line 24-25.

Objective:

Often unclearly written,

Should clearly state: "This study aimed to assess..."

Response:

Thank you very much the reviewer, the comment is accepted and corrected accordingly in manuscript from line 29-31.

Methods:

Common problems: Missing study design clarity

Poor grammar (tense inconsistency)

Lack of variable description

Improve by: Clearly stating design, sample, model, software

Response:

The methodology under abstract has improved and rewritten in manuscript from line 32-42.

Results:

Overloaded sentences

Mixing interpretation with results

Use: Key findings only

Include effect sizes (AOR, IRR, etc.)

Response:

Thank you for your valuable comments, overloaded sentences and unnecessary results were removed, clearly modified.

Conclusion:

Too generic

Not aligned with results

Should: Highlight implications

Avoid repetition

Response:

Thank you for the comment, the conclusion is now rewritten aligned with results.

4. Introduction – Detailed Review

Paragraph 1:

Grammar errors (very common)

Weak opening (too textbook-like)

Start with: Burden + significance + gap

Response:

Thank you very much for your comment. The introduction is rewritten and the grammar error also updated and highlighted from line 58-91.

Paragraph 2–3:

Issues: Lack of logical flow

Missing recent references

Overuse of global statistics without linking to study

Literature Review:

Not well synthesized

Listing studies instead of analyzing

➤ Improve: Show what is known vs unknown

Response:

Thank you very much, all necessary raised issues corrected in manuscript from line 66-81 .

Research Gap:

Often unclear or weak

Must clearly answer: What is missing?

Why this study matters?

Response:

Now, research gap added in manuscript and other necessary modification were made from line 82-91.

5. Methods Section

Study Design:
Not clearly stated at the beginning
Should be first sentence

Response:

Thank you the reviewer for your comment and the study design is now updated.

Study Setting:
Too brief or missing key details

Response:

Thank you very much for the comment. The study setting is now updated.

Population:
Inclusion/exclusion criteria unclear

Response:

Thank you for your valuable comments. The inclusion and exclusion criteria are updated and clearly stated.

Variables:
Poor operational definitions

Response:

Operational definitions are revised and corrected.

Some variables not justified

Response:

Thank you very much for the comment. Important variables are justified.

Data Analysis:
Major issues: Model selection not justified
No explanation of assumptions
Confusion between statistical approaches

Response:

Thank you for the comment. It is updated from line 229-237.

Statistical Model:
If advanced model used:

Needs clear justification
Needs simplification for readers

Response:

Thank you for the comment and the statistical model was justified and updated. The paragraphs are simplified for readers.

6. Results Section – Line-by-Line Comments

Descriptive Results:
Redundant reporting

Tables not well explained

Response

Thank you very much for the comment. Redundant reports are removed and revised.

Analytical Results:
Interpretation mixed with results
Some inconsistencies in reported values

Response:

Thank you very much, inconsistencies in reported values revised and the results are removed.

Tables:
Problems:
Missing reference categories
Poor formatting
Not self-explanatory

Response:

Thank you the reviewer for your comments. All tables are updated to the new format, self explanatory and reference categories included.

7. Discussion Section – Detailed Review

Opening Paragraph:
Should summarize key findings clearly

Response:

Thank you very much for the comment. The opening paragraph is now updated, and the key findings are clearly summarized from line 292-296.

Interpretation:
 Major issue:
Repetition of results instead of interpretation

Response:

Thank you very much the reviewer for invaluable comments. The comment is accepted and updated within the manuscript.

Comparison with Literature:
 Weak integration
 Missing critical discussion

Response:

Thank you so much for the comment. The literatures were revised and integrated with our study findings.
Explanations:
 Some are speculative without evidence

Response:

All the discussion component of the manuscript was updated and discussed based on the findings.

Policy Implication:
 Often weak or missing

Response:

The comment is accepted and added on the recommendation component of the manuscript.

Reference style(citation) use plos one standard like [,,]

Response:

Thank you for the comment. All reference styles are updated.

8. Strengths and Limitations
Strengths:
Usually acceptable but:
 Overstated

Response:

Thank you the reviewer for the comment and it is accepted.

Limitations:
 Missing key issues:
Cross-sectional limitation
Bias (recall, selection)
Missing variables

Response:

Thank you very much for the comment, the limitation of the study is now revised accordingly.

9. Conclusion
 Issues: Too long
Repetitive
Not focused
 Improve:
Keep concise
Emphasize: Key finding
Implication
Recommendation

Response

Thank you very much for the comment. The conclusion is now revised accordingly.

Reviewer #4:

Abstract
The abstract is more informative after revision, but it still feels slightly overloaded with statistical detail. A clearer balance between prevalence figures and the practical implications would make it more digestible.

Response:

Thank you so much the reviewer. The abstract is now abstract updated.

Introduction
The introduction sets the stage but could be more concise. Some background details are repeated, and the rationale for focusing on Gondar could be sharpened to emphasize the local gap in evidence.

Response:

Thank you very much for the comment and the introduction is revised according to your valuable comments.

Methods

The retrospective design is appropriate for prevalence estimation, but it inherently limits causal inference. Reliance on chart review means important socio-economic variables are missing, which weakens contextual interpretation. The sample size calculation is explained, though reviewers rightly noted that including all 719 cases might have been stronger.

Response:

Thank you the reviewer for the comment. Some of the comments are putted in the limitation of the study. However, we do not want to include all the 719 cases because if we include all cases, it could become a report instead of the research. In addition, those 303 cases can also represent the whole 719 cases.

Results

The results are presented clearly, but the heavy reliance on percentages without deeper narrative makes them feel somewhat mechanical. The tables are improved, yet some demographic variables are incomplete due to missing records, which should be acknowledged more prominently.

Response:

Thank you so much the comment and we acknowledged your comment.

Discussion

The discussion compares findings with other Ethiopian and international studies, but the explanations for differences remain somewhat superficial. More exploration of mechanisms (e.g., why primigravidity or meconium-stained fluid increase risk) would strengthen interpretation. The distinction between complications due to OVD versus underlying labor issues is important but could be emphasized further.

Response:

Thank you for the comment. The discussion part is now revised and more explanation was given specially on why primigravidity and meconium-stained fluid increase the risks.

Limitations

The limitations are appropriately acknowledged, but they could be expanded to include the potential for misclassification bias in chart reviews and the lack of long-term neonatal follow-up.

Response:

Thank you very much, limitation of the study is now updated accordingly within manuscript.

Conclusions & Recommendations

The conclusions are consistent with the findings, but the recommendations lean toward general obstetric practice improvements rather than being tightly linked to the study's specific results. For example, encouraging vacuum use is reasonable, but the study did not directly compare neonatal outcomes between vacuum and forceps in detail.

Response:

Thank you very much the reviewer for your valuable comment and the conclusion and recommendations components are now revised and updated according to your valuable comments.

Overall

The paper provides useful local evidence, but the retrospective design, single-center scope, and missing socio-economic data limit generalizability. The commentary highlights that while prevalence and risk factors are well-documented, the explanatory depth in discussion and the specificity of recommendations could be improved.

Response:

Thank you so much for the comment. We acknowledged the limitations of the study and the recommendations are now revised and specific to the concerned bodied.

Thank you very much the reviewers for your valuable comments. Much appreciated. If you have any other comments, please let us know.
