# Supplementary material for: Adverse neonatal outcomes and associated factors among births through operative vaginal delivery at the University of Gondar Comprehensive Specialized Hospital, Northwest Ethiopia: A facility-based retrospective cross-sectional study
Source: PLoS One. 2026 Aug 28;21(8):e0357043. doi: 10.1371/journal.pone.0357043 (PMC13524286; doi:10.1371/journal.pone.0357043)
Supplement: Supplementary file 2 [file pone.0357043.s001.docx]

|  | Hospital | Specific Ward | Data collector |
| --- | --- | --- | --- |
| ID (code) |  |  |  |
| Date of data collection_____________ | | | |

| **Patient identification code: _________________** | | | |
| --- | --- | --- | --- |
| 1.Obstetrics characteristics | | | |
| s. n | Questions | Response and code | Skip Code |
| 1 | Gravidity | 1. Primigravida 2. multigravida | A1 |
| 2 | parity | 1. 1-2 2. >2 | A2 |
| 3 | Gestational age by LMP, Urine HCG or early US in WKS | 1. 34-37 2. <37 3. 37-42 4. >42 | A3 |
| 4 | ANC follow up | yes  no | A4 |
| 5 | Number of ANC follow up | one visit  two to three visits  four and above visit  No visit | A5 |
| 6 | State of current pregnancy | 1. Planned 2. Unplanned | A6 |
| 7 | History of abortion | 1. Yes 2. No | A7 |
| 8 | History of child death | 1. Yes 2. No | A8 |
| 9 | Mode of previous delivery | 1. Vaginal 2. C section | A9 |
| 10 | Status of cervical dilatation at presentation | 1. Fully dilated 2. Not fully dilated 3. Closed | A10 |
| 11 | Status of fetal membrane at  presentation | 1. Intact 2. Ruptured | A11 |
| 12 | Status of liquor | clear  G1MSAF  G2MSAF  G3MSAF | A12 |

| **2.Socio-demographic characteristics** | | | |
| --- | --- | --- | --- |
| ***S.n.*** | ***Questions*** | ***Responses and Codes*** | ***Code*** |
| 1. | Residence | 1. Urban 2. Rural | B1 |
| 2. | Age in completed years | 1. --------------------years | B2 |
| 3. | Current marital status | 1. Single 2. Married 3. Divorced 4. Separated 5. Widowed | B3 |
| 4. | Educational status | 1. Unable to read and write 2. Primary school (1-8) 3. Secondary school (9-12) 4. Technical and vocational 5. Diploma 6. First degree and above   Other (specify | B4 |
| 5. | Occupational status | 1. Housewife 2. Civil servant (government employee) 3. Student 4. Farmer 5. Merchant 6. Daily laborer 7. Others (specify) | B5 |

| Part C. Fetal condition | | | |
| --- | --- | --- | --- |
| s. n | ***Questions*** | ***Responses and Codes*** | ***Code*** |
| 1. | Fetal outcome of delivery within first minute | 1.Alive  2. Dead | C1 |
| 2. | Condition of alive fetus within first five minutes | 1. Health looking  2. With birth injury  3. Asphyxiated | C2 |
| 3. | Indication | 1. Prolonged SSOL  2. NRFHRP  3. maternal indication | C3 |
| 4. | Apgar score of the newborn within first 5 minutes | 1.0  2.<7  3.>7 | C4 |
| 5. | Weight of newborn in(gm) | 1500-2499  2500-3999  >/=4000 | C5 |
| 6. | Type of OVD used | 1.Vacuum  2. Forceps | C6 |
| 7. | Type of OVD applied | 1.low  2. outlet | C7 |
| 8. | Time of application | 1. on arrival  2. followed | C8 |
| 9. | Who attended the delivery | 1. midwife  2. R1  3. R2  4. R3  5. R4  6. Senior | C9 |
| 10. | Duration of second stage | 1. <2hours  2. >2hours | C10 |
| 11. | Episiotomy | 1.Yes  2. No | C11 |
| 12. | Station | 1.+1 and above  2.+2 and below | C12 |
| 13. | Birth injury | 1. yes  1. No | C13 |
| 14. | Referred to NICU | 1.Yes  2.No | C14 |
| 15. | Resuscitated | 1.Yes  2.No | C15 |
| 16. | Maternal complication | 1.Yes  2.No | C16 |
| 17. | Type of complication e | 1.PPH  2.Laceration  3.Incontinence | C17 |
