# Supplementary material for: Adverse neonatal outcomes and associated factors among births through operative vaginal delivery at the University of Gondar Comprehensive Specialized Hospital, Northwest Ethiopia: A facility-based retrospective cross-sectional study
Source: PLoS One. 2026 Aug 28;21(8):e0357043. doi: 10.1371/journal.pone.0357043 (PMC13524286; doi:10.1371/journal.pone.0357043)
Supplement: Supplementary file 3 [file pone.0357043.s004.docx]

**Response to Reviewers**

**Reviewer #1:**

1. The abstract lacks sufficient detail regarding specific adverse outcomes assessed.

**Response:**

Thank you, the reviewer, for your valuable comment, we have revised the abstract and added sufficient details of the adverse neonatal outcomes assessed in the study and highlighted from line 40-51.

2. The abstract does not adequately state the main limitations of the study

**Response:**

Thank you very much the reviewer for the comment. We have reviewed many articles related to this research and it didn’t state the limitations of the study in the abstract section of the article.
3. The introduction does not clearly identify a specific research gap

**Response:**

We have revised the introduction to clearly articulate the research gap in the manuscript according to your comments highlighted from line 88-93.
4. There is repetition in the introduction regarding the incidence and risks of operative vaginal deliveries, making the section unnecessarily verbose

**Response:**

Thank you for your comments, repeated incidence and risks of OVD was removed within the manuscript.
5. The introduction fails to articulate a strong rationale for why this study is needed at this time and setting.

**Response:**

Thank you for your constructive comments, the rationale for why this study is needed is rewritten in the manuscript and highlighted from line 93-98.
6. Key definitions (e.g., "adverse neonatal outcomes") are not operationalized early

**Response:**

Thank you the reviewer for your valuable comment. The adverse neonatal outcome is now operationalized within the manuscript (operational definition section) and highlighted from line 210-212 as follows.

**Adverse neonatal outcomes of operational vaginal delivery**: Is when the neonate developed complications like low APGAR score, need of resuscitation, admission to NICU, neonatal birth trauma and neonatal death.

7. The literature cited in the introduction is not consistently up-to-date

**Response:**

Thank you for the comment. We updated the introduction and discussion sections by incorporating more recent literatures.
8. Retrospective cross-sectional design limit’s ability to infer causality between exposures and outcomes.

**Response:**

We acknowledged the limitation at the limitation of the study sestion. A statement clarifying that causal inference cannot be established due to the retrospective cross-sectional design has been added to the limitations section.
9. The paper does not address or adjust for all potential confounders that could influence neonatal outcomes

**Response:**

Thank you for your comments, all potential confounders that could influence neonatal outcomes managed by multivariable analysis model from line 225-232 and clearly stated from line 287-303.
10. Arbitrary p-value (<0.2) threshold for variable inclusion in multivariable analysis may introduce bias in the model selection process

**Response:**

Dear reviewer, thank you so much for the comment. It was due to typographical error, we say <0.2, but the reality present in our work is the p-value <0.25 threshold was used to select more variables from bivariable analysis.
11. Some key variables are insufficiently analyzed or discussed for their influence on neonatal outcomes

**Response:**

Necessary variables in manuscript were discussed after your comments and corrected accordingly at the discussion section of the manuscript.
12. Discussion is superficial and lacks depth, particularly regarding alternative explanations for findings

**Response**
We have strengthened the discussion by exploring alternative explanations for findings and comparing results with evidence from different articles.
13. Conclusions and recommendations are weakly supported by the presented data

**Response:**

Thank you for the comment and the conclusions and recommendations are explained within manuscript highlighted from line 378-396.
14. The generalizability of results is limited as the study is based on data from a single hospital.

**Response:**

We acknowledge this comment as the limitation of the study from line 377-378.
15. The justification for sample size and chosen margin of error is not thoroughly explained.

**Response:**

The clear justification of sample size was explained within manuscript from line 129-144, and 4% margin of error was chosen to achieve a high degree of precision and increase sample size and decrease sampling error. If margin of error selected as 5%, the final sample size of the study was becoming 194.
16. There are multiple grammatical errors and awkward phrasing throughout, which affect the quality and readability of the manuscript

**Response:**

As much as possible now, we tried to minimize the grammar errors and awkward phrases within the manuscript.
17. References include outdated sources and do not sufficiently integrate the latest evidence or guidelines relevant to the topic

**Response:**

Thank you for your comments, all outdated references are now updated
18. There are inconsistencies in terminology throughout the text (e.g., “operative vaginal delivery,” “instrumental delivery,” “OVD”)

**Response:**

In reality, operative vaginal delivery has the same meaning with instrumental delivery. However, for clarity for readers, we consistently used operational vaginal delivery throughout the manuscript.
19. Abbreviations are sometimes introduced without clear definitions or used inconsistently in the tables and narrative

**Response:**

We acknowledged the reviewer comment and now abbreviations are used in the first word of the manuscript.
20. Tables lack uniform formatting and, in some cases, do not fully explain categories or provide denominators for percentages, making interpretation difficult

**Response:**

We acknowledged the reviewer comment and now the tables are formatted as standard, the categories are fully explained by providing percentages for denominators.
21. Reference formatting is inconsistent and several citations are incomplete or do not follow journal style

**Response:**

We acknowledged the reviewer comments, and all the references are now consistent and follow the journal style.
22. There are occasional issues with alignment and spacing in tables and text

**Response:**

Thank you the reviewer for your comment and the alignment and spacing in tables and text are now the same which is 1.5 spacing.
23. Section headings are sometimes unclear or not appropriately differentiated from the main text

**Response:**

Thank you for the comment. The headings are now unclear and appropriately differentiated from the main text.
24. Some sentences are excessively long and should be broken up for clarity

**Response:**

Thank you for the comment, all necessary modifications are made accordingly.

**Reviewer #2:** 
Abstract
1. Background: this section is too long. Please reduce it.

**Response:**

Thank you the reviewer for the comment. The background section is now reduced and updated.
2. The explanation about statistical methods in abstract is too much, please reduce them and replace them with inclusion/exclusion criteria and other necessary information.

**Response:**

We acknowledged the comment. The statistical details are now reduced, and essential methodological information are included.
3. Results: what were the adverse neonatal outcomes?

**Response:**

Adverse neonatal outcome is considered when the neonate experiences one or more from the following complications: low APGAR score, need of resuscitation, retinal hemorrhage, admission to NICU, neonatal birth trauma and neonatal death
4. The most common indication for operative vaginal delivery was a prolonged second stage.. should be mentioned in the results section.

**Response:**

According to your comment, indication…prolonged second stage is stated in the results section from line 40-42.
Introduction
1. Line 71: n Ethiopia, it accounts from 13%- 42.1% in different study: it should be in the different studies.

**Response:**

We acknowledged the comment. Different study is changed to different studies within manuscript on line 67.
Methods
1. Line 139: one “formula” at the end of sentence should be deleted.

**Response:**

Thank you for the comment, correction was made accordingly.
2. You assessed all instrumental deliveries happened in two years, therefore, there is no need to formula for sample size calculation. Why you did not recruit all operational deliveries during two years?

**Response:**

Thank you for the comment. We were not assessed all instrumental deliveries happened in two years. Among 19,634 deliveries recorded over two years, 719 were delivered by operative vaginal delivery. Assessing all 719 births requires many resources and much time. It becomes a wastage if we assessed all instrumental deliveries happened in two years. Therefore, we use a sampling method to represent others.

3. How did you check the validity of questionnaire?

**Response:**

The questionnaire was validated through a pilot study conducted at the nearby Health Center to check for the validity of questionnaire and highlighted within the manuscript from line 173-174.

4. Line 188: what do you mean about favorable?

**Response:**

Thank you for the comment. We saw it unnecessary and removed from the manuscript.
5. Lines 190-197: please write each word in full before using its abbreviation.

**Response:**

The comment is acknowledged and updated.
6. The person who conduct the operational delivery is so important (resident or gynecologist, or midwife), please report.

**Response:**

Thank you for the comment. The person who conducts operational vaginal delivery were midwives, gynaecologists and emergency surgeons and clearly mentioned in table 2 under result section.
7. Line 206: it should be in the first and fifth minute after birth.

**Response:**

Thank you, we correct as the first and fifth minute after birth.
8. I understand that data collected retrospectively, please write about procedure and type of document (hard copy or in the file) and also if needed more information, did you call the women?

**Response:**

We clarified that data were collected retrospectively from patient medical records (from charts), mentioned on line 177. No direct contact with mothers was made.
Results
1. Table 1: beside of age and resident area, other demographic factors such as education, age of husband, economic status are important factors that are missing from this table.

**Response:**

Some demographic factors such as education is added, however variables such as age of husband, economic status were missed variables from medical records. Thes are the limitation of using retrospective medical records.

2. The health provider that applied instrumental delivery should be mentioned in “Table 2”.

**Response:**

Thank you for your comment, the health provider that applied instrumental delivery is now added in table 2.
Discussion
1. The analysis of situation in the specific hospital for sample collection has been not written by authors. May specific condition exist in your area compared to other areas e.g. more tendency of gynecologist for using instruments in time of delivery.

**Response:**

The comment is acknowledged. Thank you for the comment.
References
1. I noticed that most of references that even they are not new do not have the issue number and page numbers.

**Response:**

Thank you the reviewer, all the references are now updated and includes number and page numbers.

**Thank you so much the reviewers for your invaluable insights and comments. We really appreciate you.**
