# Supplementary material for: Adverse neonatal outcomes and associated factors among births through operative vaginal delivery at the University of Gondar Comprehensive Specialized Hospital, Northwest Ethiopia: A facility-based retrospective cross-sectional study
Source: PLoS One. 2026 Aug 28;21(8):e0357043. doi: 10.1371/journal.pone.0357043 (PMC13524286; doi:10.1371/journal.pone.0357043)
Supplement: Peer review history

# Peer review: Decision Letter 0

**DOI:** 10.1371/journal.pone.0357043.r001

**Authors:** Kahsu Gebrekidan

16 Feb 2026

Dear Dr. Ayele,

Thank you for submitting your manuscript to PLOS ONE. After careful consideration, we feel that it has merit but does not fully meet PLOS ONE’s publication criteria as it currently stands. Therefore, we invite you to submit a revised version of the manuscript that addresses the points raised during the review process.

Please submit your revised manuscript by Apr 02 2026 11:59PM. If you will need more time than this to complete your revisions, please reply to this message or contact the journal office at plosone@plos.org. When you're ready to submit your revision, log on to https://www.editorialmanager.com/pone/ and select the 'Submissions Needing Revision' folder to locate your manuscript file.

- A letter that responds to each point raised by the academic editor and reviewer(s). You should upload this letter as a separate file labeled 'Response to Reviewers'.
- A marked-up copy of your manuscript that highlights changes made to the original version. You should upload this as a separate file labeled 'Revised Manuscript with Track Changes'.
- An unmarked version of your revised paper without tracked changes. You should upload this as a separate file labeled 'Manuscript'.

If you would like to make changes to your financial disclosure, please include your updated statement in your cover letter. Guidelines for resubmitting your figure files are available below the reviewer comments at the end of this letter.

If applicable, we recommend that you deposit your laboratory protocols in protocols.io to enhance the reproducibility of your results. Protocols.io assigns your protocol its own identifier (DOI) so that it can be cited independently in the future. For instructions see: https://journals.plos.org/plosone/s/submission-guidelines#loc-laboratory-protocols. Additionally, PLOS ONE offers an option for publishing peer-reviewed Lab Protocol articles, which describe protocols hosted on protocols.io. Read more information on sharing protocols at https://plos.org/protocols?utm_medium=editorial-email&utm_source=authorletters&utm_campaign=protocols.

We look forward to receiving your revised manuscript.

Kind regards,

Kahsu Gebrekidan, Ph.D.

Academic Editor

PLOS One

Journal Requirements:

When submitting your revision, we need you to address these additional requirements.

1. Please ensure that your manuscript meets PLOS ONE's style requirements, including those for file naming. The PLOS ONE style templates can be found at

https://journals.plos.org/plosone/s/file?id=wjVg/PLOSOne_formatting_sample_main_body.pdf and

https://journals.plos.org/plosone/s/file?id=ba62/PLOSOne_formatting_sample_title_authors_affiliations.pdf

2. Please provide additional details regarding participant consent. In the ethics statement in the Methods and online submission information, please ensure that you have specified (1) whether consent was informed and (2) what type you obtained (for instance, written or verbal, and if verbal, how it was documented and witnessed). If your study included minors, state whether you obtained consent from parents or guardians. If the need for consent was waived by the ethics committee, please include this information.

If you are reporting a retrospective study of medical records or archived samples, please ensure that you have discussed whether all data were fully anonymized before you accessed them and/or whether the IRB or ethics committee waived the requirement for informed consent. If patients provided informed written consent to have data from their medical records used in research, please include this information.

3. Thank you for stating the following in the Competing Interests section:

I have read the journal's policy and the authors of this manuscript have the following competing interests. The authors declare that they have no competing interests.

Please confirm that this does not alter your adherence to all PLOS ONE policies on sharing data and materials, by including the following statement: "This does not alter our adherence to PLOS ONE policies on sharing data and materials.” (as detailed online in our guide for authors http://journals.plos.org/plosone/s/competing-interests). If there are restrictions on sharing of data and/or materials, please state these. Please note that we cannot proceed with consideration of your article until this information has been declared.

Please include your updated Competing Interests statement in your cover letter; we will change the online submission form on your behalf.

4. Your ethics statement should only appear in the Methods section of your manuscript. If your ethics statement is written in any section besides the Methods, please move it to the Methods section and delete it from any other section. Please ensure that your ethics statement is included in your manuscript, as the ethics statement entered into the online submission form will not be published alongside your manuscript.

5. We note that your Data Availability Statement is currently as follows: All relevant data are within the manuscript and its Supporting Information files.

Please confirm at this time whether or not your submission contains all raw data required to replicate the results of your study. Authors must share the “minimal data set” for their submission. PLOS defines the minimal data set to consist of the data required to replicate all study findings reported in the article, as well as related metadata and methods (https://journals.plos.org/plosone/s/data-availability#loc-minimal-data-set-definition).

For example, authors should submit the following data:

- The values behind the means, standard deviations and other measures reported;

- The values used to build graphs;

- The points extracted from images for analysis.

Authors do not need to submit their entire data set if only a portion of the data was used in the reported study.

If your submission does not contain these data, please either upload them as Supporting Information files or deposit them to a stable, public repository and provide us with the relevant URLs, DOIs, or accession numbers. For a list of recommended repositories, please see https://journals.plos.org/plosone/s/recommended-repositories.

If there are ethical or legal restrictions on sharing a de-identified data set, please explain them in detail (e.g., data contain potentially sensitive information, data are owned by a third-party organization, etc.) and who has imposed them (e.g., an ethics committee). Please also provide contact information for a data access committee, ethics committee, or other institutional body to which data requests may be sent. If data are owned by a third party, please indicate how others may request data access.

6. Please ensure that you refer to Figure 1 in your text as, if accepted, production will need this reference to link the reader to the figure.

7. If the reviewer comments include a recommendation to cite specific previously published works, please review and evaluate these publications to determine whether they are relevant and should be cited. There is no requirement to cite these works unless the editor has indicated otherwise.

[Note: HTML markup is below. Please do not edit.]

Reviewers' comments:

Reviewer's Responses to Questions

**Comments to the Author**

1. Is the manuscript technically sound, and do the data support the conclusions?

Reviewer #1: Partly

Reviewer #2: Yes

**********

2. Has the statistical analysis been performed appropriately and rigorously?

Reviewer #1: Yes

Reviewer #2: Yes

**********

3. Have the authors made all data underlying the findings in their manuscript fully available?

The PLOS Data policy

Reviewer #1: Yes

Reviewer #2: Yes

**********

4. Is the manuscript presented in an intelligible fashion and written in standard English?

Reviewer #1: Yes

Reviewer #2: Yes

**********

Reviewer #1: The abstract lacks sufficient detail regarding specific adverse outcomes assessed

The abstract does not adequately state the main limitations of the study

The introduction does not clearly identify a specific research gap

There is repetition in the introduction regarding the incidence and risks of operative vaginal deliveries, making the section unnecessarily verbose

The introduction fails to articulate a strong rationale for why this study is needed at this time and setting.

Key definitions (e.g., "adverse neonatal outcomes") are not operationalized early

The literature cited in the introduction is not consistently up-to-date

Retrospective cross-sectional design limits ability to infer causality between exposures and outcomes

The paper does not address or adjust for all potential confounders that could influence neonatal outcomes

Arbitrary p-value (<0.2) threshold for variable inclusion in multivariable analysis may introduce bias in the model selection process

Some key variables are insufficiently analyzed or discussed for their influence on neonatal outcomes

Discussion is superficial and lacks depth, particularly regarding alternative explanations for findings

See the following papers

Abuhammad, S., Mukattash, T. L., Alazzam, S. I., Yafawi, R., Jarab, A. S., Nusair, M. B., ... & Alrabadi, N. N. (2021). Caesarean section delivery from maternal perspective: An exploratory study in Jordan. International Journal of Clinical Practice, 75(8), e14349.

Hatamleh, R., Abujilban, S., Al-Shraideh, A. J., & Abuhammad, S. (2019). Maternal request for cesarian birth without medical indication in a group of healthy women: A qualitative study in Jordan. Midwifery, 79, 102543.

Conclusions and recommendations are weakly supported by the presented data

The generalizability of results is limited as the study is based on data from a single hospital.

The justification for sample size and chosen margin of error is not thoroughly explained.

There are multiple grammatical errors and awkward phrasing throughout, which affect the quality and readability of the manuscript

References include outdated sources and do not sufficiently integrate the latest evidence or guidelines relevant to the topic

There are inconsistencies in terminology throughout the text (e.g., “operative vaginal delivery,” “instrumental delivery,” “OVD”) t

Abbreviations are sometimes introduced without clear definitions or used inconsistently in the tables and narrative

Tables lack uniform formatting and in some cases do not fully explain categories or provide denominators for percentages, making interpretation difficult

Reference formatting is inconsistent and several citations are incomplete or do not follow journal style

There are occasional issues with alignment and spacing in tables and text

Section headings are sometimes unclear or not appropriately differentiated from the main text.

Some sentences are excessively long and should be broken up for clarity

Reviewer #2: Thank you as you invited me to review this manuscript. Please see my comments as follows:

Abstract

1. Background: this section is too long. Please reduce it.

2. The explanation about statistical methods in abstract is too much, please reduce them and replace them with inclusion/exclusion criteria and other necessary information.

3. Results: what were the adverse neonatal outcomes?

4. The most common indication for operative vaginal delivery was a prolonged second stage.. should be mentioned in the results section.

Introduction

1. Line 71: n Ethiopia, it accounts from 13%- 42.1% in different study: it should be in the different studies.

Methods

1. Line 139: one “formula” at the end of sentence should be deleted.

2. You assessed all instrumental deliveries happened in two years, therefore, there is no need to formula for sample size calculation. Why you did not recruit all operational deliveries during two years?

3. How did you check the validity of questionnaire?

4. Line 188: what do you mean about favorable?

5. Lines 190-197: please write each word in full before using its abbreviation.

6. The person who conduct the operational delivery is so important (resident or gynecologist, or midwife), please report.

7. Line 206: it should be in the first and fifth minute after birth.

8. I understand that data collected retrospectively, please write about procedure and type of document (hard copy or in the file) and also if needed more information, did you call the women?

Results

1. Table 1: beside of age and resident area, other demographic factors such as education, age of husband, economic status are important factors that are missing from this table.

2. The health provider that applied instrumental delivery should be mentioned in “Table 2”.

Discussion

1. The analysis of situation in the specific hospital for sample collection has been not written by authors. May specific condition exist in your area compared to other areas e.g. more tendency of gynecologist for using instruments in time of delivery.

References

1. I noticed that most of references that even they are not new do not have the issue number and page numbers.

**********

what does this mean?). If published, this will include your full peer review and any attached files.

If you choose “no”, your identity will remain anonymous but your review may still be made public.

**Do you want your identity to be public for this peer review?**  For information about this choice, including consent withdrawal, please see our Privacy Policy

Reviewer #1: No

Reviewer #2: **Yes:**  Prof Parvin Abedi

**********

[NOTE: If reviewer comments were submitted as an attachment file, they will be attached to this email and accessible via the submission site. Please log into your account, locate the manuscript record, and check for the action link "View Attachments". If this link does not appear, there are no attachment files.]

To ensure your figures meet our technical requirements, please review our figure guidelines: https://journals.plos.org/plosone/s/figures

You may also use PLOS’s free figure tool, NAAS, to help you prepare publication quality figures: https://journals.plos.org/plosone/s/figures#loc-tools-for-figure-preparation.

NAAS will assess whether your figures meet our technical requirements by comparing each figure against our figure specifications.

# Author comment: Author response to Decision Letter 1

**DOI:** 10.1371/journal.pone.0357043.r002

15 Mar 2026

Response to Reviewers

Reviewer #1:

1. The abstract lacks sufficient detail regarding specific adverse outcomes assessed.

Response:

Thank you, the reviewer, for your valuable comment, we have revised the abstract and added sufficient details of the adverse neonatal outcomes assessed in the study and highlighted from line 39-49.

2. The abstract does not adequately state the main limitations of the study

Response:

Thank you very much the reviewer for the comment. We have reviewed many articles related to this research and it didn’t state the limitations of the study in the abstract section of the article.

3. The introduction does not clearly identify a specific research gap

Response:

We have revised the introduction to clearly articulate the research gap in the manuscript according to your comments highlighted from line 87-92.

4. There is repetition in the introduction regarding the incidence and risks of operative vaginal deliveries, making the section unnecessarily verbose

Response:

Thank you for your comments, repeated incidence and risks of OVD was removed within the manuscript.

5. The introduction fails to articulate a strong rationale for why this study is needed at this time and setting.

Response:

Thank you for your constructive comments, the rationale for why this study is needed is rewritten in the manuscript and highlighted from line 92-97.

6. Key definitions (e.g., "adverse neonatal outcomes") are not operationalized early

Response:

Thank you the reviewer for your valuable comment. The adverse neonatal outcome is now operationalized within the manuscript (operational definition section) and highlighted from line 208-210 as follows.

Adverse neonatal outcomes of operational vaginal delivery: Is when the neonate developed complications like low APGAR score, need of resuscitation, admission to NICU, neonatal birth trauma and neonatal death.

7. The literature cited in the introduction is not consistently up-to-date

Response:

Thank you for the comment. We updated the introduction and discussion sections by incorporating more recent literatures.

8. Retrospective cross-sectional design limit’s ability to infer causality between exposures and outcomes.

Response:

We acknowledged the limitation at the limitation of the study sestion. A statement clarifying that causal inference cannot be established due to the retrospective cross-sectional design has been added to the limitations section.

9. The paper does not address or adjust for all potential confounders that could influence neonatal outcomes

Response:

Thank you for your comments, all potential confounders that could influence neonatal outcomes managed by multivariable analysis model from line 225-232 and clearly stated from line 287-297.

10. Arbitrary p-value (<0.2) threshold for variable inclusion in multivariable analysis may introduce bias in the model selection process

Response:

Dear reviewer, thank you so much for the comment. It was due to typographical error, we say <0.2, but the reality present in our work is the p-value <0.25 threshold was used to select more variables from bivariable analysis.

11. Some key variables are insufficiently analyzed or discussed for their influence on neonatal outcomes

Response:

Necessary variables in manuscript were discussed after your comments and corrected accordingly at the discussion section of the manuscript.

12. Discussion is superficial and lacks depth, particularly regarding alternative explanations for findings

Response

We have strengthened the discussion by exploring alternative explanations for findings and comparing results with evidence from different articles.

13. Conclusions and recommendations are weakly supported by the presented data

Response:

Thank you for the comment and the conclusions and recommendations are explained within manuscript highlighted from line 378-395.

14. The generalizability of results is limited as the study is based on data from a single hospital.

Response:

We acknowledge this comment as the limitation of the study from line 376-377.

15. The justification for sample size and chosen margin of error is not thoroughly explained.

Response:

The clear justification of sample size was explained within manuscript from line 129-144, and 4% margin of error was chosen to achieve a high degree of precision and increase sample size and decrease sampling error. If margin of error selected as 5%, the final sample size of the study was becoming 194.

16. There are multiple grammatical errors and awkward phrasing throughout, which affect the quality and readability of the manuscript

Response:

As much as possible now, we tried to minimize the grammar errors and awkward phrases within the manuscript.

17. References include outdated sources and do not sufficiently integrate the latest evidence or guidelines relevant to the topic

Response:

Thank you for your comments, all outdated references are now updated

18. There are inconsistencies in terminology throughout the text (e.g., “operative vaginal delivery,” “instrumental delivery,” “OVD”)

Response:

In reality, operative vaginal delivery has the same meaning with instrumental delivery. However, for clarity for readers, we consistently used operational vaginal delivery throughout the manuscript.

19. Abbreviations are sometimes introduced without clear definitions or used inconsistently in the tables and narrative

Response:

We acknowledged the reviewer comment and now abbreviations are used in the first word of the manuscript.

20. Tables lack uniform formatting and, in some cases, do not fully explain categories or provide denominators for percentages, making interpretation difficult

Response:

We acknowledged the reviewer comment and now the tables are formatted as standard, the categories are fully explained by providing percentages for denominators.

21. Reference formatting is inconsistent and several citations are incomplete or do not follow journal style

Response:

We acknowledged the reviewer comments, and all the references are now consistent and follow the journal style.

22. There are occasional issues with alignment and spacing in tables and text

Response:

Thank you the reviewer for your comment and the alignment and spacing in tables and text are now the same which is 1.5 spacing.

23. Section headings are sometimes unclear or not appropriately differentiated from the main text

Response:

Thank you for the comment. The headings are now unclear and appropriately differentiated from the main text.

24. Some sentences are excessively long and should be broken up for clarity

Response:

Thank you for the comment, all necessary modifications are made accordingly.

Reviewer #2:

Abstract

1. Background: this section is too long. Please reduce it.

Response:

Thank you the reviewer for the comment. The background section is now reduced and updated.

2. The explanation about statistical methods in abstract is too much, please reduce them and replace them with inclusion/exclusion criteria and other necessary information.

Response:

We acknowledged the comment. The statistical details are now reduced, and essential methodological information are included.

3. Results: what were the adverse neonatal outcomes?

Response:

Adverse neonatal outcome is considered when the neonate experiences one or more from the following complications: low APGAR score, need of resuscitation, retinal hemorrhage, admission to NICU, neonatal birth trauma and neonatal death

4. The most common indication for operative vaginal delivery was a prolonged second stage.. should be mentioned in the results section.

Response:

According to your comment, indication…prolonged second stage is stated in the results section from line 41-43.

Introduction

1. Line 71: n Ethiopia, it accounts from 13%- 42.1% in different study: it should be in the different studies.

Response:

We acknowledged the comment. Different study is changed to different studies within manuscript on line 65.

Methods

1. Line 139: one “formula” at the end of sentence should be deleted.

Response:

Thank you for the comment, correction was made accordingly.

2. You assessed all instrumental deliveries happened in two years, therefore, there is no need to formula for sample size calculation. Why you did not recruit all operational deliveries during two years?

Response:

Thank you for the comment. We were not assessed all instrumental deliveries happened in two years. Among 19,634 deliveries recorded over two years, 719 were delivered by operative vaginal delivery. Assessing all 719 births requires many resources and much time. It becomes a wastage if we assessed all instrumental deliveries happened in two years. Therefore, we use a sampling method to represent others.

3. How did you check the validity of questionnaire?

Response:

The questionnaire was validated through a pilot study conducted at the nearby Health Center to check for the validity of questionnaire and highlighted within the manuscript from line 172-173.

4. Line 188: what do you mean about favorable?

Response:

Thank you for the comment. We saw it unnecessary and removed from the manuscript.

5. Lines 190-197: please write each word in full before using its abbreviation.

Response:

The comment is acknowledged and updated.

6. The person who conduct the operational delivery is so important (resident or gynecologist, or midwife), please report.

Response:

Thank you for the comment. The person who conducts operational vaginal delivery were midwives, gynaecologists and emergency surgeons and clearly mentioned in table 2 under result section.

7. Line 206: it should be in the first and fifth minute after birth.

Response:

Thank you, we correct as the first and fifth minute after birth.

8. I understand that data collected retrospectively, please write about procedure and type of document (hard copy or in the file) and also if needed more information, did you call the women?

Response:

We clarified that data were collected retrospectively from patient medical records (from charts), mentioned on line 177. No direct contact with mothers was made.

Results

1. Table 1: beside of age and resident area, other demographic factors such as education, age of husband, economic status are important factors that are missing from this table.

Response:

Some demographic factors such as education is added, however variables such as age of husband, economic status were missed variables from medical records. Thes are the limitation of using retrospective medical records.

2. The health provider that applied instrumental delivery should be mentioned in “Table 2”.

Response:

Thank you for your comment, the health provider that applied instrumental delivery is now added in table 2.

Discussion

1. The analysis of situation in the specific hospital for sample collection has been not written by authors. May specific condition exist in your area compared to other areas e.g. more tendency of gynecologist for using instruments in time of delivery.

Response:

The comment is acknowledged. Thank you for the comment.

References

1. I noticed that most of references that even they are not new do not have the issue number and page numbers.

Response:

Thank you the reviewer, all the references are now updated and includes number and page numbers.

Thank you so much the reviewers for your invaluable insights and comments. We really appreciate you.

Attachment Submitted filename: Response to Reviewers.docx

# Peer review: Decision Letter 1

**DOI:** 10.1371/journal.pone.0357043.r003

**Authors:** Kahsu Gebrekidan

1 Jun 2026

Dear Dr. Ayele,

Thank you for submitting your manuscript to PLOS ONE. After careful consideration, we feel that it has merit but does not fully meet PLOS ONE’s publication criteria as it currently stands. Therefore, we invite you to submit a revised version of the manuscript that addresses the points raised during the review process.

Please submit your revised manuscript by Jul 16 2026 11:59PM**.**  If you will need more time than this to complete your revisions, please reply to this message or contact the journal office at plosone@plos.org. When you're ready to submit your revision, log on to https://www.editorialmanager.com/pone/ and select the 'Submissions Needing Revision' folder to locate your manuscript file.

- A letter that responds to each point raised by the academic editor and reviewer(s). You should upload this letter as a separate file labeled 'Response to Reviewers'.
- A marked-up copy of your manuscript that highlights changes made to the original version. You should upload this as a separate file labeled 'Revised Manuscript with Track Changes'.
- An unmarked version of your revised paper without tracked changes. You should upload this as a separate file labeled 'Manuscript'.

If you would like to make changes to your financial disclosure, please include your updated statement in your cover letter. Guidelines for resubmitting your figure files are available below the reviewer comments at the end of this letter.

If applicable, we recommend that you deposit your laboratory protocols in protocols.io to enhance the reproducibility of your results. Protocols.io assigns your protocol its own identifier (DOI) so that it can be cited independently in the future. For instructions see: https://journals.plos.org/plosone/s/submission-guidelines#loc-laboratory-protocols. Additionally, PLOS ONE offers an option for publishing peer-reviewed Lab Protocol articles, which describe protocols hosted on protocols.io. Read more information on sharing protocols at https://plos.org/protocols?utm_medium=editorial-email&utm_source=authorletters&utm_campaign=protocols.

As the corresponding author, your ORCID iD is verified in the submission system and will appear in the published article. PLOS supports the use of ORCID, and we encourage all coauthors to register for an ORCID iD and use it as well. Please encourage your coauthors to verify their ORCID iD within the submission system before final acceptance, as unverified ORCID iDs will not appear in the published article. *Only*  the individual author can complete the verification step; PLOS staff *cannot*  verify ORCID iDs on behalf of authors.

We look forward to receiving your revised manuscript.

Kind regards,

Kahsu Gebrekidan, Ph.D.

Academic Editor

PLOS One

Journal Requirements:

If the reviewer comments include a recommendation to cite specific previously published works, please review and evaluate these publications to determine whether they are relevant and should be cited. There is no requirement to cite these works unless the editor has indicated otherwise.

[Note: HTML markup is below. Please do not edit.]

Reviewers' comments:

Reviewer's Responses to Questions

**Comments to the Author**

Reviewer #3: All comments have been addressed

Reviewer #4: (No Response)

**********

2. Is the manuscript technically sound, and do the data support the conclusions?

Reviewer #3: Partly

Reviewer #4: No

**********

3. Has the statistical analysis been performed appropriately and rigorously?

Reviewer #3: No

Reviewer #4: I Don't Know

**********

4. Have the authors made all data underlying the findings in their manuscript fully available?

The PLOS Data policy

Reviewer #3: No

Reviewer #4: No

**********

5. Is the manuscript presented in an intelligible fashion and written in standard English?

Reviewer #3: No

Reviewer #4: Yes

**********

Reviewer #3: Abstract

Background:

❌ Too general or lacks quantitative context

Add specific statistics and not write its burden in % in abstract part

Objective:

❌ Often unclearly written,

Should clearly state: “This study aimed to assess…”

Methods:

❌ Common problems: Missing study design clarity

Poor grammar (tense inconsistency)

Lack of variable description

Improve by: Clearly stating design, sample, model, software

Results:

❌ Overloaded sentences

❌ Mixing interpretation with results

Use:Key findings only

Include effect sizes (AOR, IRR, etc.)

Conclusion:

❌ Too generic

❌ Not aligned with results

Should: Highlight implications

Avoid repetition

4. Introduction – Detailed Review

Paragraph 1:

❌ Grammar errors (very common)

❌ Weak opening (too textbook-like)

Start with: Burden + significance + gap

Paragraph 2–3:

❌ Issues: Lack of logical flow

Missing recent references

Overuse of global statistics without linking to study

Literature Review:

❌ Not well synthesized

❌ Listing studies instead of analyzing

➤ Improve: Show what is known vs unknown

Research Gap:

❌ Often unclear or weak

Must clearly answer: What is missing?

Why this study matters?

5. Methods Section

Study Design:

❌ Not clearly stated at the beginning

Should be first sentence

Study Setting:

❌ Too brief or missing key details

Population:

❌ Inclusion/exclusion criteria unclear

Variables:

❌ Poor operational definitions

❌ Some variables not justified

Data Analysis:

❌ Major issues: Model selection not justified

No explanation of assumptions

Confusion between statistical approaches

Statistical Model:

❌ If advanced model used:

Needs clear justification

Needs simplification for readers

6. Results Section – Line-by-Line Comments

Descriptive Results:

❌ Redundant reporting

❌ Tables not well explained

Analytical Results:

❌ Interpretation mixed with results

❌ Some inconsistencies in reported values

Tables:

❌ Problems:

Missing reference categories

Poor formatting

Not self-explanatory

7. Discussion Section – Detailed Review

Opening Paragraph:

❌ Should summarize key findings clearly

Interpretation:

❌ Major issue:

Repetition of results instead of interpretation

Comparison with Literature:

❌ Weak integration

❌ Missing critical discussion

Explanations:

❌ Some are speculative without evidence

Policy Implication:

❌ Often weak or missing

Reference style(citation) use plos one standard like [,,]

8. Strengths and Limitations

Strengths:

Usually acceptable but:

❌ Overstated

Limitations:

❌ Missing key issues:

Cross-sectional limitation

Bias (recall, selection)

Missing variables

9. Conclusion

❌ Issues: Too long

Repetitive

Not focused

✅ Improve:

Keep concise

Emphasize: Key finding

Implication

Recommendation

Final Recommendation

MAJOR REVISION

Reviewer #4: Abstract

The abstract is more informative after revision, but it still feels slightly overloaded with statistical detail. A clearer balance between prevalence figures and the practical implications would make it more digestible.

Introduction

The introduction sets the stage but could be more concise. Some background details are repeated, and the rationale for focusing on Gondar could be sharpened to emphasize the local gap in evidence.

Methods

The retrospective design is appropriate for prevalence estimation, but it inherently limits causal inference. Reliance on chart review means important socio‑economic variables are missing, which weakens contextual interpretation. The sample size calculation is explained, though reviewers rightly noted that including all 719 cases might have been stronger.

Results

The results are presented clearly, but the heavy reliance on percentages without deeper narrative makes them feel somewhat mechanical. The tables are improved, yet some demographic variables are incomplete due to missing records, which should be acknowledged more prominently.

Discussion

The discussion compares findings with other Ethiopian and international studies, but the explanations for differences remain somewhat superficial. More exploration of mechanisms (e.g., why primigravidity or meconium‑stained fluid increase risk) would strengthen interpretation. The distinction between complications due to OVD versus underlying labor issues is important but could be emphasized further.

Limitations

The limitations are appropriately acknowledged, but they could be expanded to include the potential for misclassification bias in chart reviews and the lack of long‑term neonatal follow‑up.

Conclusions & Recommendations

The conclusions are consistent with the findings, but the recommendations lean toward general obstetric practice improvements rather than being tightly linked to the study’s specific results. For example, encouraging vacuum use is reasonable, but the study did not directly compare neonatal outcomes between vacuum and forceps in detail.

Overall

The paper provides useful local evidence, but the retrospective design, single‑center scope, and missing socio‑economic data limit generalizability. The commentary highlights that while prevalence and risk factors are well‑documented, the explanatory depth in discussion and the specificity of recommendations could be improved.

**********

what does this mean?). If published, this will include your full peer review and any attached files.

If you choose “no”, your identity will remain anonymous but your review may still be made public.

**Do you want your identity to be public for this peer review?**  For information about this choice, including consent withdrawal, please see our Privacy Policy

Reviewer #3: No

Reviewer #4: No

**********

[NOTE: If reviewer comments were submitted as an attachment file, they will be attached to this email and accessible via the submission site. Please log into your account, locate the manuscript record, and check for the action link "View Attachments". If this link does not appear, there are no attachment files.]

To ensure your figures meet our technical requirements, please review our figure guidelines: https://journals.plos.org/plosone/s/figures

You may also use PLOS’s free figure tool, NAAS, to help you prepare publication quality figures: https://journals.plos.org/plosone/s/figures#loc-tools-for-figure-preparation.

NAAS will assess whether your figures meet our technical requirements by comparing each figure against our figure specifications.

# Author comment: Author response to Decision Letter 2

**DOI:** 10.1371/journal.pone.0357043.r004

13 Jun 2026

PONE-D-25-28741R1

Adverse neonatal outcomes and associated factors among births through operative vaginal delivery at the University of Gondar Comprehensive Specialized Hospital, Northwest Ethiopia: A facility based retrospective cross-sectional study

Thank you so much the editor and reviewers of your valuable comments. We saw these comments as an input for having a better manuscript.

Reviewers' comments and our responses to questions

Reviewer #3:

Abstract

Background:

Too general or lacks quantitative context

Add specific statistics and not write its burden in % in abstract part

Response:

Thank you very much the reviewer for your valuable comment. The comment is accepted and updated within the manuscript from line 24-25.

Objective:

Often unclearly written,

Should clearly state: “This study aimed to assess…”

Response:

Thank you very much the reviewer, the comment is accepted and corrected accordingly in manuscript from line 29-31.

Methods:

Common problems: Missing study design clarity

Poor grammar (tense inconsistency)

Lack of variable description

Improve by: Clearly stating design, sample, model, software

Response:

The methodology under abstract has improved and rewritten in manuscript from line 32-42.

Results:

Overloaded sentences

Mixing interpretation with results

Use: Key findings only

Include effect sizes (AOR, IRR, etc.)

Response:

Thank you for your valuable comments, overloaded sentences and unnecessary results were removed, clearly modified.

Conclusion:

Too generic

Not aligned with results

Should: Highlight implications

Avoid repetition

Response:

Thank you for the comment, the conclusion is now rewritten aligned with results.

4. Introduction – Detailed Review

Paragraph 1:

Grammar errors (very common)

Weak opening (too textbook-like)

Start with: Burden + significance + gap

Response:

Thank you very much for your comment. The introduction is rewritten and the grammar error also updated and highlighted from line 58-91.

Paragraph 2–3:

Issues: Lack of logical flow

Missing recent references

Overuse of global statistics without linking to study

Literature Review:

Not well synthesized

Listing studies instead of analyzing

➤ Improve: Show what is known vs unknown

Response:

Thank you very much, all necessary raised issues corrected in manuscript from line 66-81 .

Research Gap:

Often unclear or weak

Must clearly answer: What is missing?

Why this study matters?

Response:

Now, research gap added in manuscript and other necessary modification were made from line 82-91.

5. Methods Section

Study Design:

Not clearly stated at the beginning

Should be first sentence

Response:

Thank you the reviewer for your comment and the study design is now updated.

Study Setting:

Too brief or missing key details

Response:

Thank you very much for the comment. The study setting is now updated.

Population:

Inclusion/exclusion criteria unclear

Response:

Thank you for your valuable comments. The inclusion and exclusion criteria are updated and clearly stated.

Variables:

Poor operational definitions

Response:

Operational definitions are revised and corrected.

Some variables not justified

Response:

Thank you very much for the comment. Important variables are justified.

Data Analysis:

Major issues: Model selection not justified

No explanation of assumptions

Confusion between statistical approaches

Response:

Thank you for the comment. It is updated from line 229-237.

Statistical Model:

If advanced model used:

Needs clear justification

Needs simplification for readers

Response:

Thank you for the comment and the statistical model was justified and updated. The paragraphs are simplified for readers.

6. Results Section – Line-by-Line Comments

Descriptive Results:

Redundant reporting

Tables not well explained

Response

Thank you very much for the comment. Redundant reports are removed and revised.

Analytical Results:

Interpretation mixed with results

Some inconsistencies in reported values

Response:

Thank you very much, inconsistencies in reported values revised and the results are removed.

Tables:

Problems:

Missing reference categories

Poor formatting

Not self-explanatory

Response:

Thank you the reviewer for your comments. All tables are updated to the new format, self explanatory and reference categories included.

7. Discussion Section – Detailed Review

Opening Paragraph:

Should summarize key findings clearly

Response:

Thank you very much for the comment. The opening paragraph is now updated, and the key findings are clearly summarized from line 292-296.

Interpretation:

Major issue:

Repetition of results instead of interpretation

Response:

Thank you very much the reviewer for invaluable comments. The comment is accepted and updated within the manuscript.

Comparison with Literature:

Weak integration

Missing critical discussion

Response:

Thank you so much for the comment. The literatures were revised and integrated with our study findings.

Explanations:

Some are speculative without evidence

Response:

All the discussion component of the manuscript was updated and discussed based on the findings.

Policy Implication:

Often weak or missing

Response:

The comment is accepted and added on the recommendation component of the manuscript.

Reference style(citation) use plos one standard like [,,]

Response:

Thank you for the comment. All reference styles are updated.

8. Strengths and Limitations

Strengths:

Usually acceptable but:

Overstated

Response:

Thank you the reviewer for the comment and it is accepted.

Limitations:

Missing key issues:

Cross-sectional limitation

Bias (recall, selection)

Missing variables

Response:

Thank you very much for the comment, the limitation of the study is now revised accordingly.

9. Conclusion

Issues: Too long

Repetitive

Not focused

Improve:

Keep concise

Emphasize: Key finding

Implication

Recommendation

Response

Thank you very much for the comment. The conclusion is now revised accordingly.

Reviewer #4:

Abstract

The abstract is more informative after revision, but it still feels slightly overloaded with statistical detail. A clearer balance between prevalence figures and the practical implications would make it more digestible.

Response:

Thank you so much the reviewer. The abstract is now abstract updated.

Introduction

The introduction sets the stage but could be more concise. Some background details are repeated, and the rationale for focusing on Gondar could be sharpened to emphasize the local gap in evidence.

Response:

Thank you very much for the comment and the introduction is revised according to your valuable comments.

Methods

The retrospective design is appropriate for prevalence estimation, but it inherently limits causal inference. Reliance on chart review means important socio economic variables are missing, which weakens contextual interpretation. The sample size calculation is explained, though reviewers rightly noted that including all 719 cases might have been stronger.

Response:

Thank you the reviewer for the comment. Some of the comments are putted in the limitation of the study. However, we do not want to include all the 719 cases because if we include all cases, it could become a report instead of the research. In addition, those 303 cases can also represent the whole 719 cases.

Results

The results are presented clearly, but the heavy reliance on percentages without deeper narrative makes them feel somewhat mechanical. The tables are improved, yet some demographic variables are incomplete due to missing records, which should be acknowledged more prominently.

Response:

Thank you so much the comment and we acknowledged your comment.

Discussion

The discussion compares findings with other Ethiopian and international studies, but the explanations for differences remain somewhat superficial. More exploration of mechanisms (e.g., why primigravidity or meconium stained fluid increase risk) would strengthen interpretation. The distinction between complications due to OVD versus underlying labor issues is important but could be emphasized further.

Response:

Thank you for the comment. The discussion part is now revised and more explanation was given specially on why primigravidity and meconium stained fluid increase the risks.

Limitations

The limitations are appropriately acknowledged, but they could be expanded to include the potential for misclassification bias in chart reviews and the lack of long term neonatal follow up.

Response:

Thank you very much, limitation of the study is now updated accordingly within manuscript.

Conclusions & Recommendations

The conclusions are consistent with the findings, but the recommendations lean toward general obstetric practice improvements rather than being tightly linked to the study’s specific results. For example, encouraging vacuum use is reasonable, but the study did not directly compare neonatal outcomes between vacuum and forceps in detail.

Response:

Thank you very much the reviewer for your valuable comment and the conclusion and recommendations components are now revised and updated according to your valuable comments.

Overall

The paper provides useful local evidence, but the retrospective design, single center scope, and missing socio economic data limit generalizability. The commentary highlights that while prevalence and risk factors are well documented, the explanatory depth in discussion and the specificity of recommendations could be improved.

Response:

Thank you so much for the comment. We acknowledged the limitations of the study and the recommendations are now revised and specific to the concerned bodied.

Thank you very much the reviewers for your valuable comments. Much appreciated. If you have any other comments, please let us know.

Attachment Submitted filename: Response to Reviewers comments second round.pdf

# Peer review: Decision Letter 2

**DOI:** 10.1371/journal.pone.0357043.r005

**Authors:** Kahsu Gebrekidan

11 Aug 2026

Adverse neonatal outcomes and associated factors among births through operative vaginal delivery at the University of Gondar Comprehensive Specialized Hospital, Northwest Ethiopia: A facility based retrospective cross-sectional study

PONE-D-25-28741R2

Dear Dr. Habtamu,

We’re pleased to inform you that your manuscript has been judged scientifically suitable for publication and will be formally accepted for publication once it meets all outstanding technical requirements.

Within one week, you’ll receive an e-mail detailing the required amendments. When these have been addressed, you’ll receive a formal acceptance letter and your manuscript will be scheduled for publication.

An invoice will be generated when your article is formally accepted. Please note, if your institution has a publishing partnership with PLOS and your article meets the relevant criteria, all or part of your publication costs will be covered. Please make sure your user information is up-to-date by logging into Editorial Manager at Editorial Manager® and clicking the ‘Update My Information' link at the top of the page. For questions related to billing, please contact billing support.

If your institution or institutions have a press office, please notify them about your upcoming paper to help maximize its impact. If they’ll be preparing press materials, please inform our press team as soon as possible -- no later than 48 hours after receiving the formal acceptance. Your manuscript will remain under strict press embargo until 2 pm Eastern Time on the date of publication. For more information, please contact onepress@plos.org.

Kind regards,

Kahsu Gebrekidan, Ph.D.

Academic Editor

PLOS One

Additional Editor Comments (optional):

Reviewers' comments:

Reviewer's Responses to Questions

**Comments to the Author**

Reviewer #3: All comments have been addressed

**********

2. Is the manuscript technically sound, and do the data support the conclusions?

Reviewer #3: Yes

**********

3. Has the statistical analysis been performed appropriately and rigorously?

Reviewer #3: Yes

**********

4. Have the authors made all data underlying the findings in their manuscript fully available?

The PLOS Data policy

Reviewer #3: Yes

**********

5. Is the manuscript presented in an intelligible fashion and written in standard English?

Reviewer #3: Yes

**********

Reviewer #3: I would like to sincerely appreciate the authors for their thorough and careful revision of the manuscript. All the previously raised concerns have been adequately addressed. The revisions have significantly improved the clarity, methodological rigor, statistical presentation, and overall scientific quality of the paper. The manuscript is now well structured, and the interpretation of the findings is appropriate and consistent with the results. I commend the authors for their effort in strengthening the work.

**********

what does this mean?). If published, this will include your full peer review and any attached files.

If you choose “no”, your identity will remain anonymous but your review may still be made public.

**Do you want your identity to be public for this peer review?**  For information about this choice, including consent withdrawal, please see our Privacy Policy

Reviewer #3: No

**********

# Editor report: Acceptance letter

**DOI:** 10.1371/journal.pone.0357043.r006

**Authors:** Kahsu Gebrekidan

PONE-D-25-28741R2

PLOS One

Dear Dr. Ayele,

I'm pleased to inform you that your manuscript has been deemed suitable for publication in PLOS One. Congratulations! Your manuscript is now being handed over to our production team.

At this stage, our production department will prepare your paper for publication. This includes ensuring the following:

* All references, tables, and figures are properly cited

* All relevant supporting information is included in the manuscript submission,

* There are no issues that prevent the paper from being properly typeset

You will receive further instructions from the production team, including instructions on how to review your proof when it is ready. Please keep in mind that we are working through a large volume of accepted articles, so please give us a few days to review your paper and let you know the next and final steps.

Lastly, if your institution or institutions have a press office, please let them know about your upcoming paper now to help maximize its impact. If they'll be preparing press materials, please inform our press team within the next 48 hours. Your manuscript will remain under strict press embargo until 2 pm Eastern Time on the date of publication. For more information, please contact onepress@plos.org.

You will receive an invoice from PLOS for your publication fee after your manuscript has reached the completed accept phase. If you receive an email requesting payment before acceptance or for any other service, this may be a phishing scheme. Learn how to identify phishing emails and protect your accounts at https://explore.plos.org/phishing.

If we can help with anything else, please email us at customercare@plos.org.

Thank you for submitting your work to PLOS One and supporting open access.

Kind regards,

PLOS ONE Editorial Office Staff

on behalf of

Dr. Kahsu Gebrekidan

Academic Editor

PLOS One
